# Integrated transcriptomic and metabolomic analyses reveal phytohormone signaling mechanisms underlying differential regeneration potential in microspore-derived calli from hulled and hulless barley

**DOI:** 10.1186/s12870-026-09535-1

**Published:** 2026-07-16

**Authors:** Runhong Gao, Guimei Guo, Yingjie Zong, Wenqi Zhang, Longhua Zhou, Hongwei Xu, Yingbo Li, Chenghong Liu

**Affiliations:** Shanghai Key Laboratory of Agricultural Genetics and Breeding, Biotechnology Research Institute, Shanghai Academy of Agricultural Sciences, Shanghai, 201106 China

**Keywords:** Isolated microspore culture, Barley, Regeneration capability, Transcriptomics, Metabolomics, Phytohormone signaling

## Abstract

**Background:**

Isolated microspore culture (IMC) is an effective model for investigating plant cell totipotency and is widely used in plant breeding programs to rapidly produce doubled haploid lines. However, the limited regeneration capacity of hulless barely restricts the broader application of IMC in breeding improvement and research.

**Results:**

In this study, two hulled barley genotypes (Hua30 and L07) exhibiting high regeneration capability and two hulless barley genotypes (Zangqing2000 and Ximala22) with poor regeneration capability were selected to comparatively evaluate the regeneration potential of microspore-derived calli generated via IMC through integrated metabolomic and transcriptomic analyses. The regeneration capability of the two hulled genotypes exceeded 200 green plantlets per 100 mg microspore-derived callus, whereas that of the hulless genotypes was nearly absent. A total of 2,647 differentially expressed genes (DEGs) and 128 differentially accumulated metabolites (DAMs) were identified in microspore-derived callus from hulled genotypes relative to hulless genotypes. Integrative analysis of transcriptomic and metabolomic datasets indicated that phytohormone signaling pathways play a central role in regulating regeneration competence acquisition. Hulled barley exhibited enhanced hormonal homeostasis and comparatively lower levels of jasmonic acid (JA) and brassinosteroids (BR), which are critical determinants of regeneration capacity in microspore calli. The exogenous application of JA and BR to the induction media markedly inhibited the subsequent regeneration of plantlets in hulled barley. Several transcription factors (TFs) associated with plant regeneration, including *WOX7*,* BBM2*, and *LBD*, showed differential expression patterns between hulled and hulless barley, suggesting their potential functions in regulating regeneration capacity. In addition, the hulled/hulless caryopsis phenotype may be closely associated with the regeneration potential of the microspore-derived callus.

**Conclusions:**

These findings provide mechanistic insights into the molecular mechanisms underlying plant regeneration in barley.

**Supplementary Information:**

The online version contains supplementary material available at https://doi.org/10.1186/s12870-026-09535-1.

## Background

Isolated microspore culture (IMC) is a widely used method for generating double haploid (DH) plants, providing an efficient tool for plant breeding by rapidly producing homozygous lines [1]. Under in vitro conditions, isolated microspores can switch from the gametophytic developmental pathway to the sporophytic pathway, undergoing haploid cell division and subsequently forming embryos or calli. These calli or embryos can then be regenerated into DH plants through spontaneous or chemically induced diploidization under appropriate culture conditions [2–4]. Therefore, callus or embryo induction and DH plant regeneration represent the two critical stages of IMC [5, 6]. The efficiency of DH plant regeneration is as an important indicator of the physiological state of microspore-derived embryos or calli and reflects the overall effectiveness of the IMC system as a breeding tool [7]. Most studies on microspore embryogenesis have focused on optimizing induction techniques and elucidating the mechanisms underlying callus or embryo formation [4, 8, 9]. However, the molecular mechanism regulating DH plant regeneration via the IMC pathway remains poorly understood.

Multiple factors, including genotype, growth conditions of donor plants, microspore developmental stage, pretreatment conditions, and culture medium composition, influence the regeneration capability of DH plants from microspore-derived calli, reflecting the overall effectiveness of microspore embryogenesis [3, 10]. Among these variables, genotype dependency represents the principal limiting factor affecting DH plant regeneration during microspore embryogenesis [11].

Barley (*Hordeum vulgare* L.) is the fourth most important global cereal crop, after wheat, rice, and maize. Based on caryopsis characteristics, barley is classified into hulled and hulless (or naked) types [12]. In most domesticated cultivars, the husk adheres tightly to the grain after threshing, forming hulled barley, which is primarily used for malting and animal feed. In contrast, certain cultivars, such as highland barley cultivated on the Qinghai-Tibet Plateau, exhibit loosely attached husks that are easily removed during threshing, resulting in hulless barley, which is commonly used for human consumption due to its processing convenience and improved edibility [13, 14]. An efficient IMC system for hulled barley has been established and is widely used in germplasm development and breeding programs [15, 16]. Although IMC system has been developed for hulless barley, its application in DH breeding is severely constrained by its markedly low regeneration efficiency [17].

In this study, two hulled cultivars (Hua30 and L07) and two hulless cultivars (Zangqing2000 and Ximala22) were selected to evaluate the regeneration capacity via IMC. Integrated transcriptomic and metabolomic analyses were conducted to elucidate the differences between hulled and hulless barley in the regeneration potential of microspore-derived calli. These findings provide insights into the genetic and metabolic determinants of regeneration capacity.

## Results

### Microspore callus induction and plant regeneration in four cultivars

**Fig. 1:**
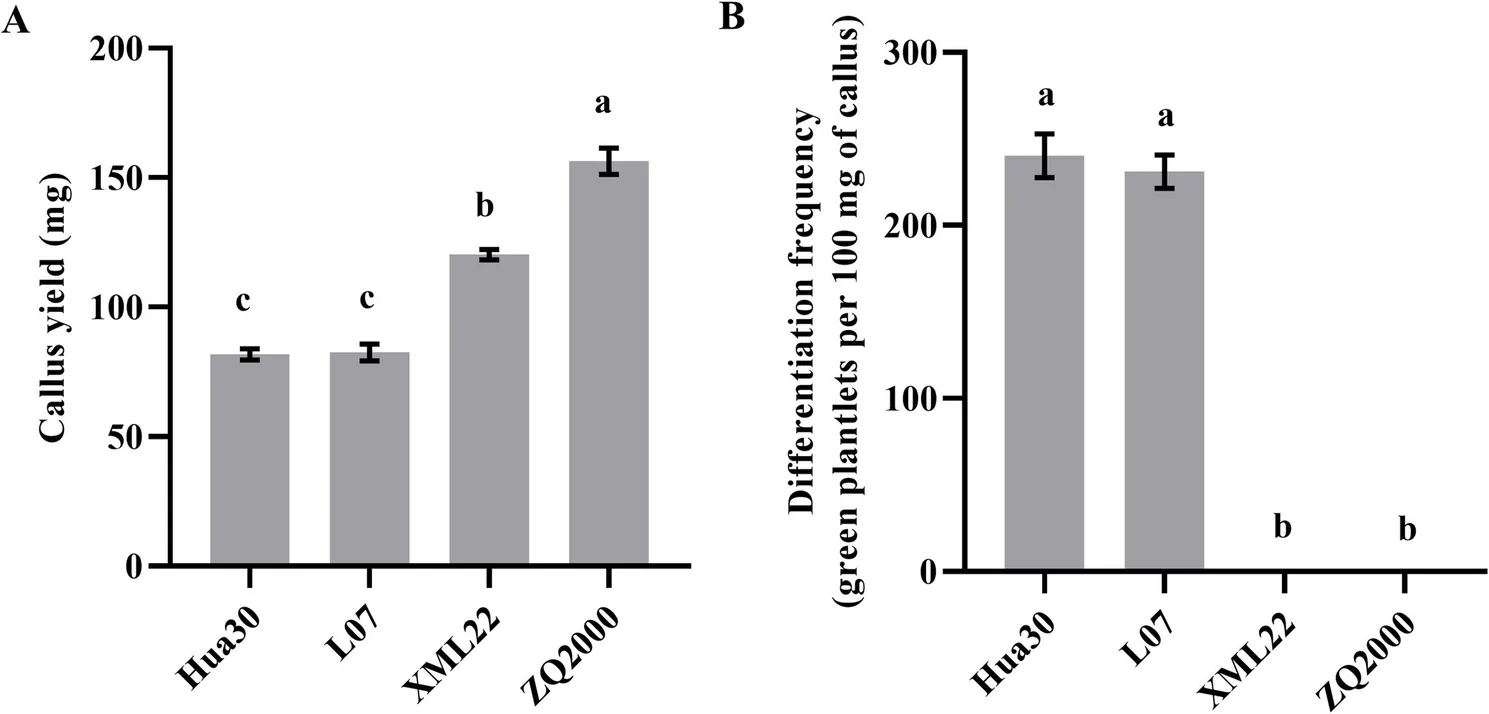
Callus yield and plant regeneration ability from four barley cultivars. Data were shown as means ± standard errors (*n* = 3). Different lowercase letters above the error bars indicate statistically significant differences based on one-way ANOVA by LSD test (*p* < 0.05)

Callus induction was assessed based on callus yield (Fig. 1A). ZQ2000 exhibited the highest callus induction ability among the four cultivars, followed by XML22. Hua30 and L07 exhibited relatively lower callus induction than ZQ2000 and XML22. Plant regeneration capacity was evaluated using differentiation frequency (Fig. 1B). The differentiation frequency of Hua30 and L07 exceeded 200 green plantlets per 100 mg of callus, whereas XML22 and ZQ2000 produced nearly no green plantlets, with differentiation frequencies approaching zero. Although microspores from hulless barley cultivars (XML22 and ZQ2000) exhibited significantly higher callus induction, their regeneration ability was markedly lower than that of hulled barley cultivars (Hua30 and L07).

### Transcriptomic analysis of DEGs in barley

**Fig. 2:**
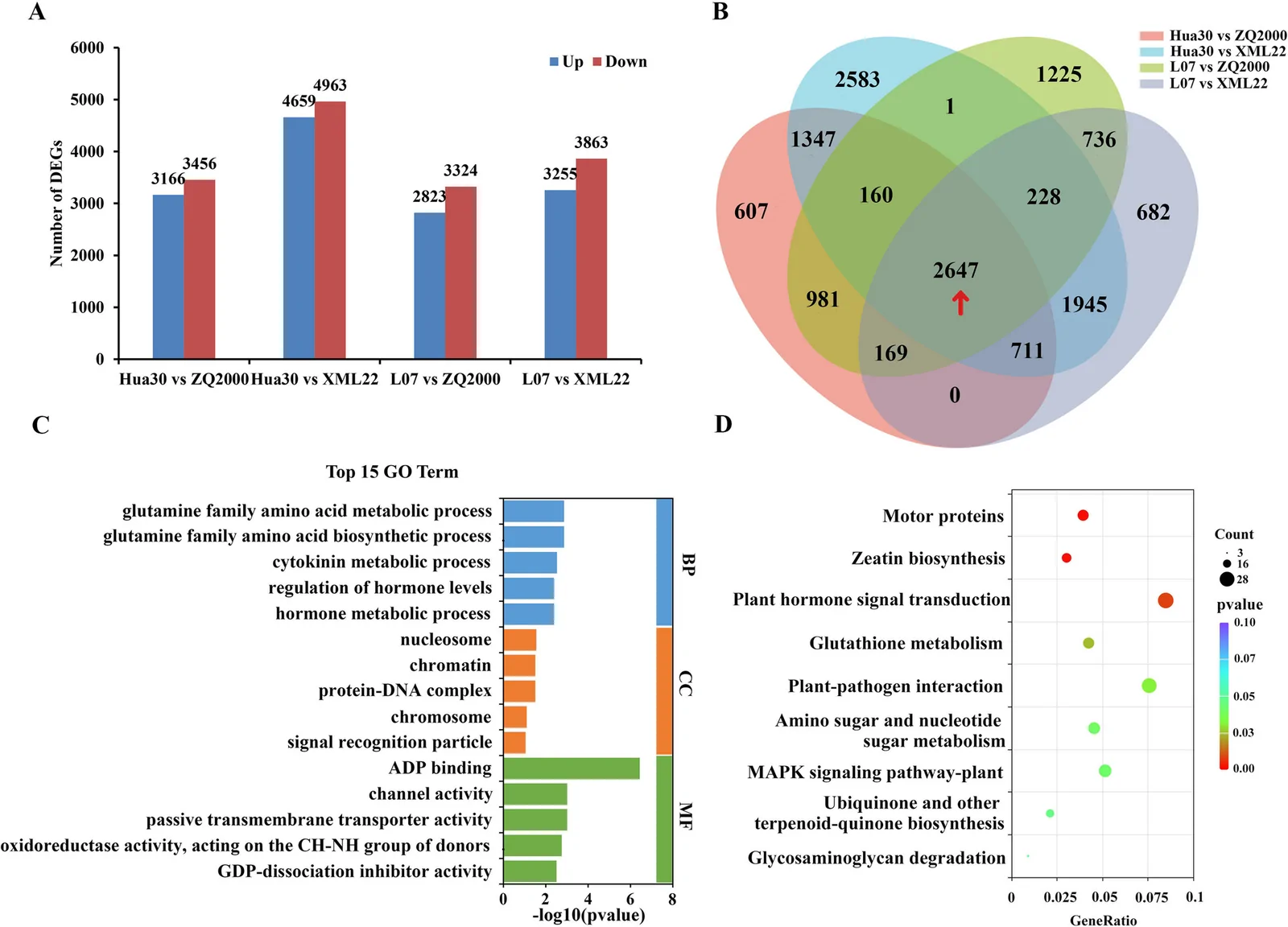
Overview of the transcriptomics analysis in barley microspore callus. **A** Number of upregulated and downregulated DEGs in four comparison groups. **B** Venn diagrams of DEGs in four comparison groups. The overlapping part (indicated by the red arrow) represents the specified DEGs both in Hua30 and L07. **C** Gene Ontology (GO) functional classification of the specified DEGs. BP: biological processes, CC: cellular component, MF: molecular function. **D** KEGG pathway enrichment of the specified DEGs

Based on the observed differences in plant regeneration ability, high-throughput RNA sequencing was performed on microspore-derived calli from Hua30, L07, XML22, and ZQ2000. To identify DEGs, pairwise comparisons were conducted between hulled and hulless barley (Hua 30 vs. ZQ2000, Hua 30 vs. XML22, L07 vs. ZQ2000, and L07 vs. XML22). Figure 2A shows the number of DEGs in each comparison. A total of 2,647 DEGs were consistently identified in all four comparisons, representing core genes potentially involved in callus differentiation in hulled barley (Fig. 2B). Functional annotations of these DEGs were conducted using GO and KEGG enrichment analyses. GO enrichment analysis identified 49 significantly enriched terms, including 23 biological processes, 3 cellular components, and 23 molecular functions. Within biological processes, DEGs were predominantly associated with glutamine family amino acid metabolic process (GO:0009064), glutamine family amino acid biosynthetic process (GO:0009084), and cytokinin metabolic process (GO:0009690). Within cellular components, DEGs were mainly enriched in nucleosomes (GO:0000786), chromatin (GO:0000785), and protein-DNA complex (GO:0032993). Within molecular functions, DEGs were primarily related to adenosine diphosphate (ADP) binding (GO:0043531), channel activity (GO:0015267), and passive transmembrane transporter activity (GO:0022803) (Fig. 2C). The KEGG enrichment analysis assigned the DEGs to 108 pathways, of which 9 were significantly enriched, including motor proteins, zeatin biosynthesis, plant hormone signal transduction, and glutathione metabolism (Fig. 2D).

### Metabolomic analysis of DAMs in barley

**Fig. 3:**
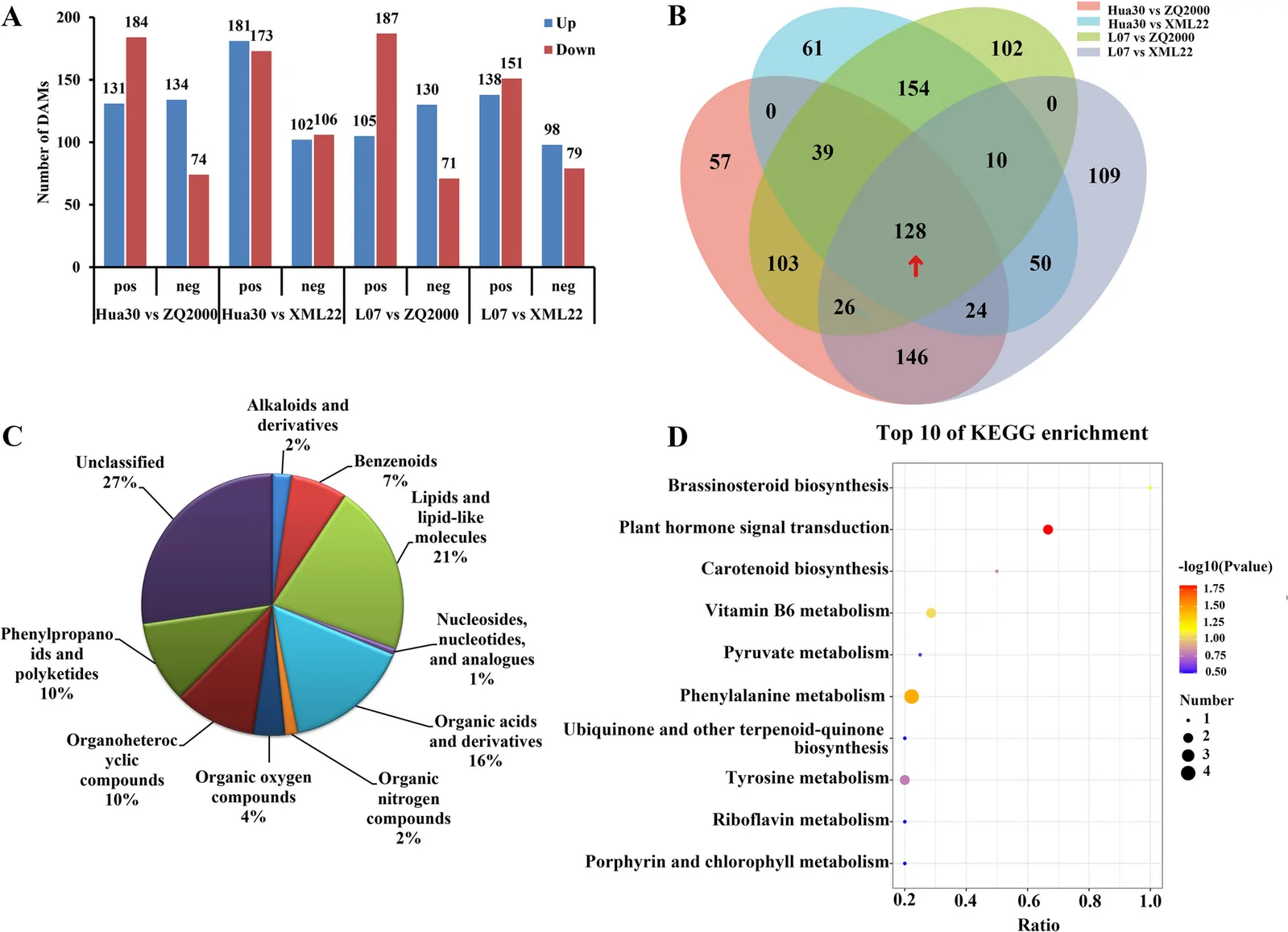
Overview of the metabolome analysis in barley microspore. **A** Number of upregulated and downregulated DAMs in four comparison groups. pos: positive ion; neg: negative ion (**B**) Venn diagrams of DAMs (pos + neg) in four comparison groups. The overlapping part (indicated by the red arrow) represents the specified DAMs both in Hua30 and L07. **C** Classification of the specified DAMs. **D** KEGG pathway enrichment of the specified DAMs

To investigate the differences in the regeneration potential of microspore-derived calli between hulled and hulless barley, metabolomic profiling was performed using UHPLC-MS/MS on the same samples used for transcriptomic analysis. Pairwise comparisons were conducted between hulled and hulless barley (Hua 30 vs. ZQ2000, Hua 30 vs. XML22, L07 vs. ZQ2000, and L07 vs. XML22). Figure 3A shows the number of DAMs identified in each comparison. A total of 128 DAMs were detected in hulled barley under positive and negative ion modes (Fig. 3B), and these were used for subsequent analyses. The DAMs were classified into 10 categories: lipids and lipid-like molecules (27), organic acids and derivatives (20), phenylpropanoids and polyketides (13), organoheterocyclic compounds (13), benzenoids (9), organic oxygen compounds (5), alkaloids and derivatives (3), organic nitrogen compounds (2), nucleosides, nucleotides, and analogues (1), and unclassified metabolites (35) (Fig. 3C). KEGG pathway enrichment analysis of DAMs detected in both positive and negative ion modes revealed significant enrichment in two pathways (*p* < 0.05): plant hormone signal transduction (map04075) and phenylalanine metabolism (map00360) (Fig. 3D).

### Changes in transcription factors

**Fig. 4:**
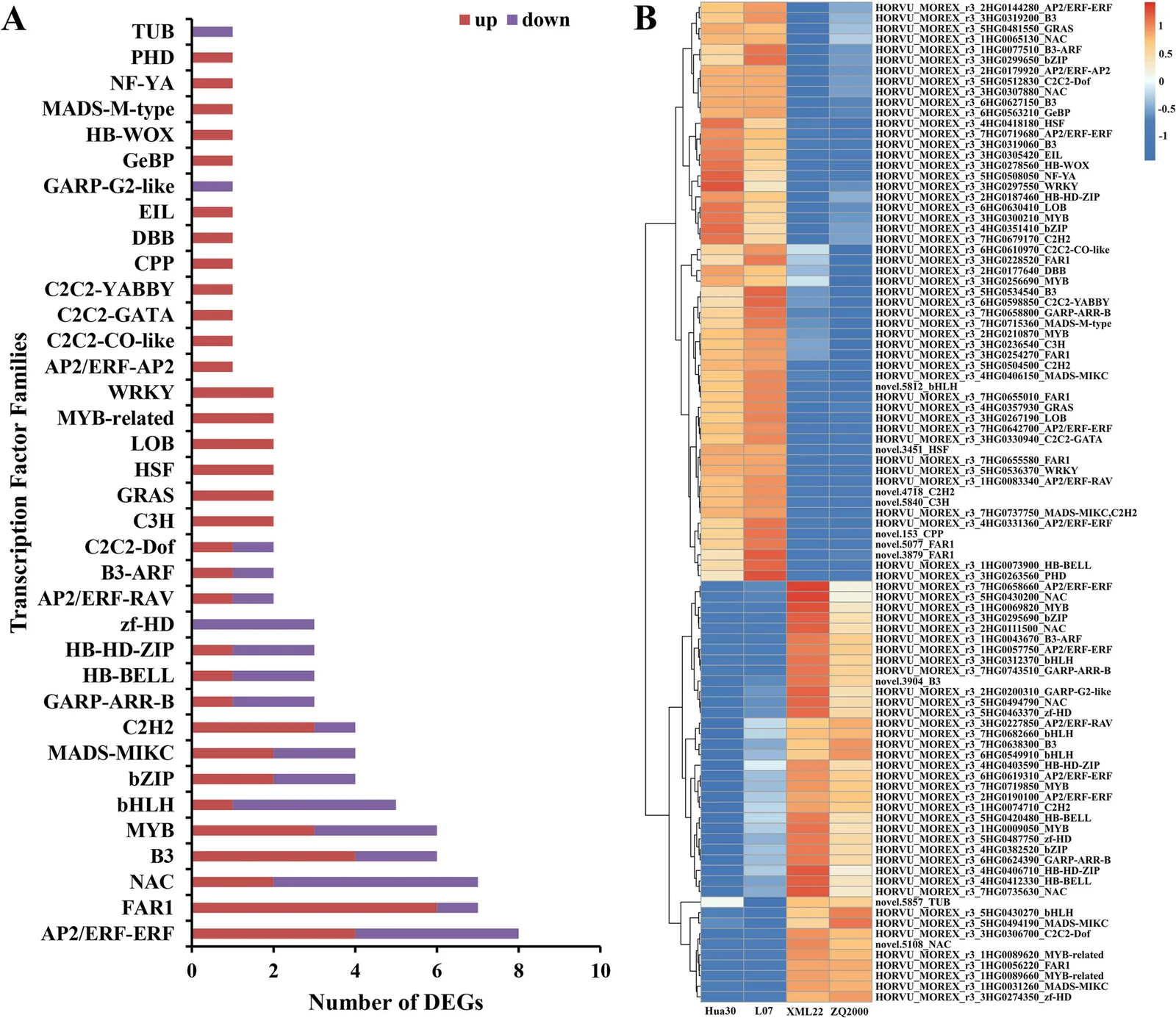
Transcription factors (TFs) differential expressed in hulled barley compared to hulless barley. **A** Number of up or down regulated gene in different TF families of hulled barley. **B** Heat-map of TF expression levels in four cultivars. The color gradient from blue to red indicates TF expression levels from low to high. Detailed information was provided in Table S1

A total of 95 transcription factors (TFs) from 36 TF families were annotated in microspore-derived calli (Fig. 4A), of which 57 were upregulated and 38 were downregulated in hulled barley (Fig. 4B, Table S1). TF families with notable expression in hulled barley included C3H, GRAS, HSF, LOB, MYB-related, WRKY, AP2/ERF-AP2, C2C2-CO-like, C2C2-GATA, C2C2-YABBY, CPP, DBB, EIL, GeBP, HB-WOX, MADS-M-type, NF-YA, and PHD. The TF families that were entirely downregulated in hulled barley included zf-HD, GARP-G2-like, and TUB. Notably, an AP2-like ethylene-responsive TF BABY BOOM 2 (BBM2) (*HORVU_MOREX_r3_2HG0179920*) and a WUSCHEL-related homeobox 7 (WOX7) (*HORVU_MOREX_r3_3HG0278560*) were significantly expressed in hulled barley. BBM and WOX are well-established regulators of plant cell totipotency. In this study, BBM2 was up-regulated by more than three-fold, and WOX7 by approximately three-fold in hulled barley compared with hulless barley, indicating their critical roles in plant regeneration of microspore-derived calli. Moreover, two lateral organ boundaries (LOB) domain proteins (*HORVU_MOREX_r3_6HG0630410* and *HORVU_MOREX_r3_3HG0267190)* were significantly upregulated in the microspore-derived callus of hulled barley. Wax inducer 1 (WIN1) *(HORVU_MOREX_r3_7HG0719680)*, an ethylene-responsive TF associated with hull-caryopsis adhesion, was upregulated more than seven-fold in hulled barley compared with hulless barley.

### Combined transcriptome and metabolome analysis

**Fig. 5:**
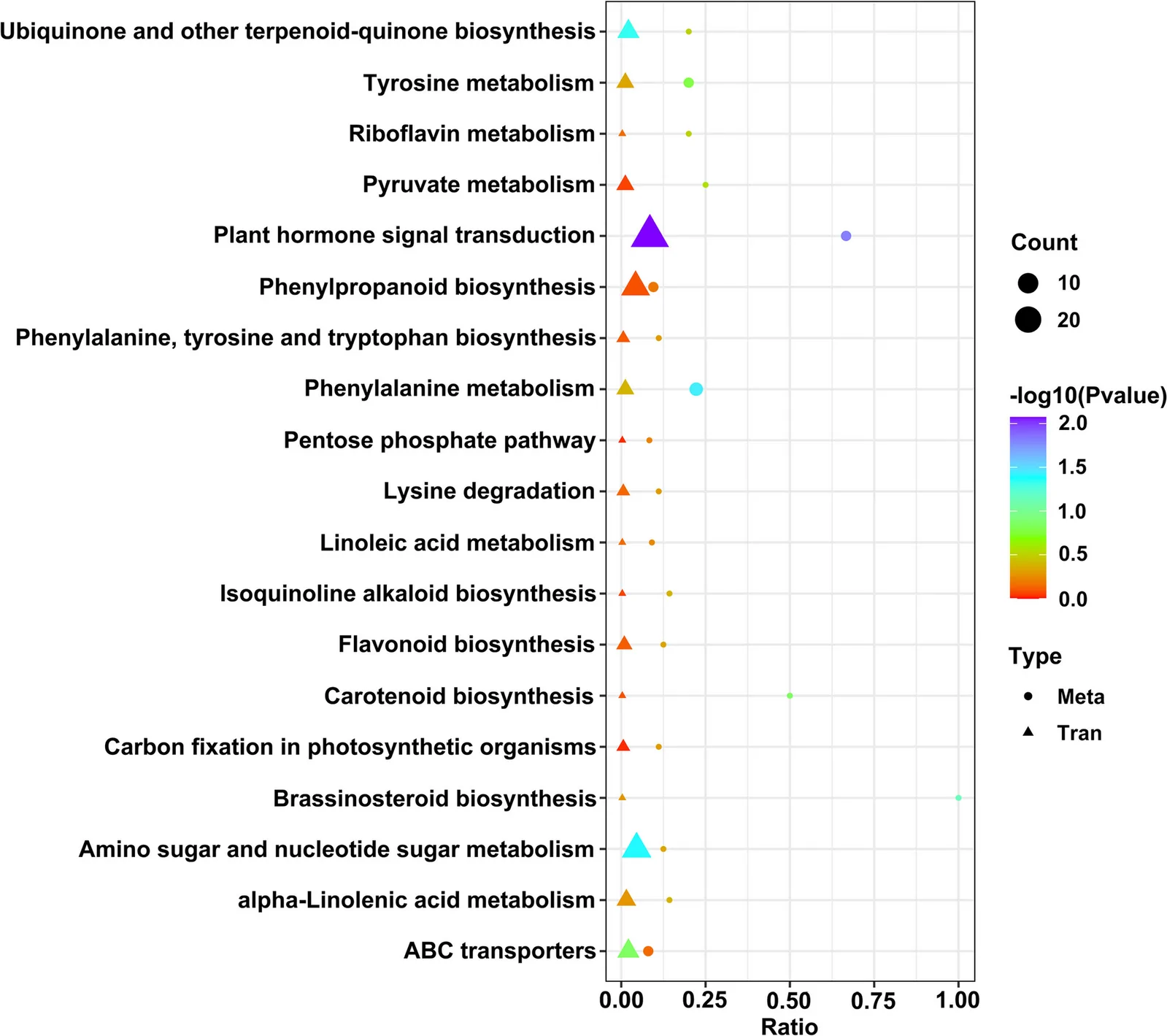
Combined analysis of transcriptome and metabolome. KEGG enrichment pathways in specified DAMs and DEGs

Integrated analysis of transcriptomic and metabolomic data revealed that plant hormone signal transduction was the only KEGG pathway that was significantly enriched. Additionally, alpha-linolenic acid metabolism and brassinosteroid biosynthesis were selected for further analysis due to significant changes in JA and BR levels (Fig. 5).

**Fig. 6:**
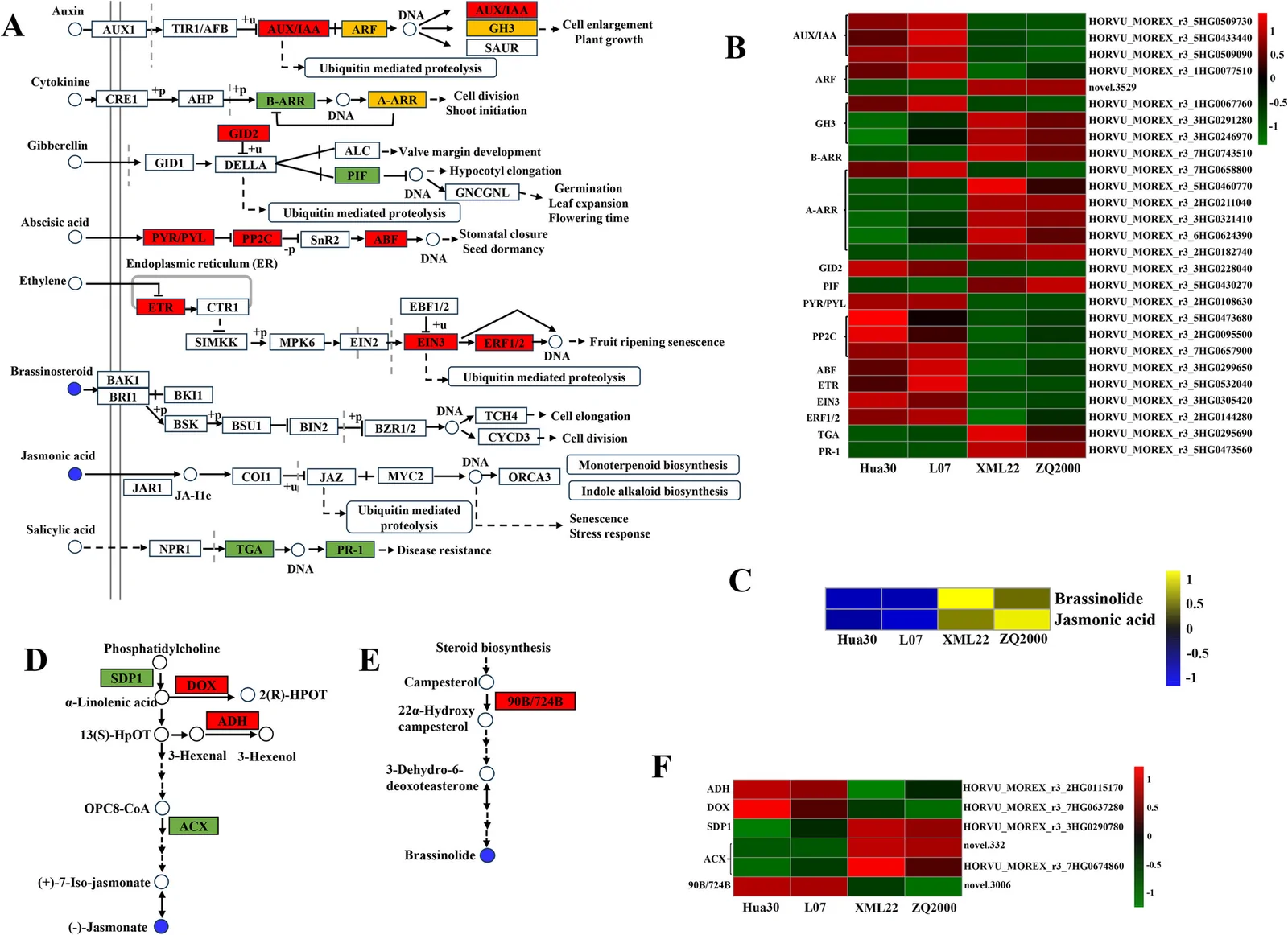
Integrated analysis of the identified DEGs and differentially accumulated plant hormone. **A** Plant hormone signal transduction pathway. Red boxes means up regulated DEGs, green boxes means down regulated DEGs, and the orange boxes means up and down regulated DEGs. The blue circles means down-regulated DAMs. The same as below. **B** Heat-map of DEGs involved in phytohormone signaling pathway. The color gradient from green to red indicates DEG expression levels from low to high. Detailed information was provided in Table S2. **C** Heat-map of DAMs involved in phytohormone. Blue-to-yellow gradient shows low-to-high relative content and accumulation of DAMs. Detailed information was provided in Table S3. **D** α-Linolenic acid metabolism pathway. **E** Brassinosteroid biosynthesis pathway. **F** Heat-map of DEGs involved in α-Linolenic acid metabolism pathway and Brassinosteroid biosynthesis pathway. Green-to-red color gradient shows low -to-high expression of DEGs. Detailed information was provided in Table S4

Within the plant hormone signal transduction pathway, 27 DEGs encoding 15 enzymes were identified. Three *auxin/indole-3-acetic acid* (*AUX/IAA*) genes (*HORVU_MOREX_r3_5HG0509730*,* HORVU_MOREX_r3_5HG0433440*,* HORVU_MOREX_r3_5HG0509090*), one *auxin response factor* (*ARF*) gene (*HORVU_MOREX_r3_1HG0077510*), and one *Gretchen Hagen 3* (*GH3*) gene (*HORVU_MOREX_r3_1HG0067760*) were significantly upregulated in the auxin (IAA) signaling pathway, whereas one *ARF* gene (*novel.3529*) and two *GH3* genes (*HORVU_MOREX_r3_3HG0246970*,* HORVU_MOREX_r3_3HG0291280*) were downregulated in hulled barley. In the cytokinin (CK) signaling pathway, one *type-B Arabidopsis response regulator* (*B-ARR*) gene (*HORVU_MOREX_r3_7HG0743510*) and five *type-A ARR* (*A-ARR*) genes (*HORVU_MOREX_r3_6HG0624390*,* HORVU_MOREX_r3_2HG0182740*,* HORVU_MOREX_r3_2HG0211040*,* HORVU_MOREX_r3_5HG0460770*, and *HORVU_MOREX_r3_3HG0321410*) were downregulated, whereas one *A-ARR* gene (*HORVU_MOREX_r3_7HG0658800*) was upregulated in hulled barley. In the gibberellin (GA) signaling pathway, one *gibberellin-insensitive dwarf 2* (*GID2*) gene (*HORVU_MOREX_r3_3HG0228040*) was upregulated, whereas one *phytochrome-interacting factor (PIF)* gene (*HORVU_MOREX_r3_5HG0430270*) was downregulated. Five DEGs associated with *pyrabactin resistance 1-like* (*PYR/PYL*) (*HORVU_MOREX_r3_2HG0108630*), *protein phosphatase 2 C (PP2C)* (*HORVU_MOREX_r3_5HG0473680*,* HORVU_MOREX_r3_2HG0095500*,* HORVU_MOREX_r3_7HG0657900*), and *abscisic acid -responsive element-binding factor (ABF)* (*HORVU_MOREX_r3_3HG0299650*) were upregulated in the abscisic acid (ABA) signaling pathway. In the ethylene (ETH) signaling pathway, three DEGs–*ethylene receptor* (*ETR*) (*HORVU_MOREX_r3_5HG0532040*), *ethylene insensitive 3* (*EIN3*) (*HORVU_MOREX_r3_3HG0305420*), and *ethylene response factor 1/2* (*ERF1/2*) (*HORVU_MOREX_r3_2HG0144280*)–were significantly up-regulated in hulled barley. DEGs were not detected in the BR and JA signaling pathways. In the salicylic acid (SA) signaling pathway, one *TGACG-Binding* (*TGA*) gene (*HORVU_MOREX_r3_3HG0295690*) and one *pathogenesis-related protein 1* (*PR-1*) gene (*HORVU_MOREX_r3_5HG0473560*) were notably downregulated in hulled barley (Fig. 6A and B). Metabolomic analysis showed that BR and JA, accumulated at lower levels in hulled barley than in hulless barley (Fig. 6C).

In the alpha-linolenic acid metabolism pathway, hulled barley had lower JA levels than hulless barley. Five DEGs encoding four enzymes were identified in this pathway. One *alcohol dehydrogenase* (*ADH*) gene (*HORVU_MOREX_r3_2HG0115170*) and one *alpha-dioxygenase* (*DOX*) gene (*HORVU_MOREX_r3_7HG0637280*) were upregulated in hulled barley. Two *acyl-CoA oxidase* (*ACX*) genes (*novel.332* and *HORVU_MOREX_r3_7HG0674860*), and one *triacylglycerol lipase* 1 (*SDP1*) gene (*HORVU_MOREX_r3_3HG0290780*) were downregulated in hulled barley. In the BR biosynthesis pathway, only one *steroid 22 S-hydroxylase* (*90B/724B*) gene was upregulated in hulled barley (Figs. 6D-F).

### Verification of DEGs by qRT- PCR

**Fig. 7:**
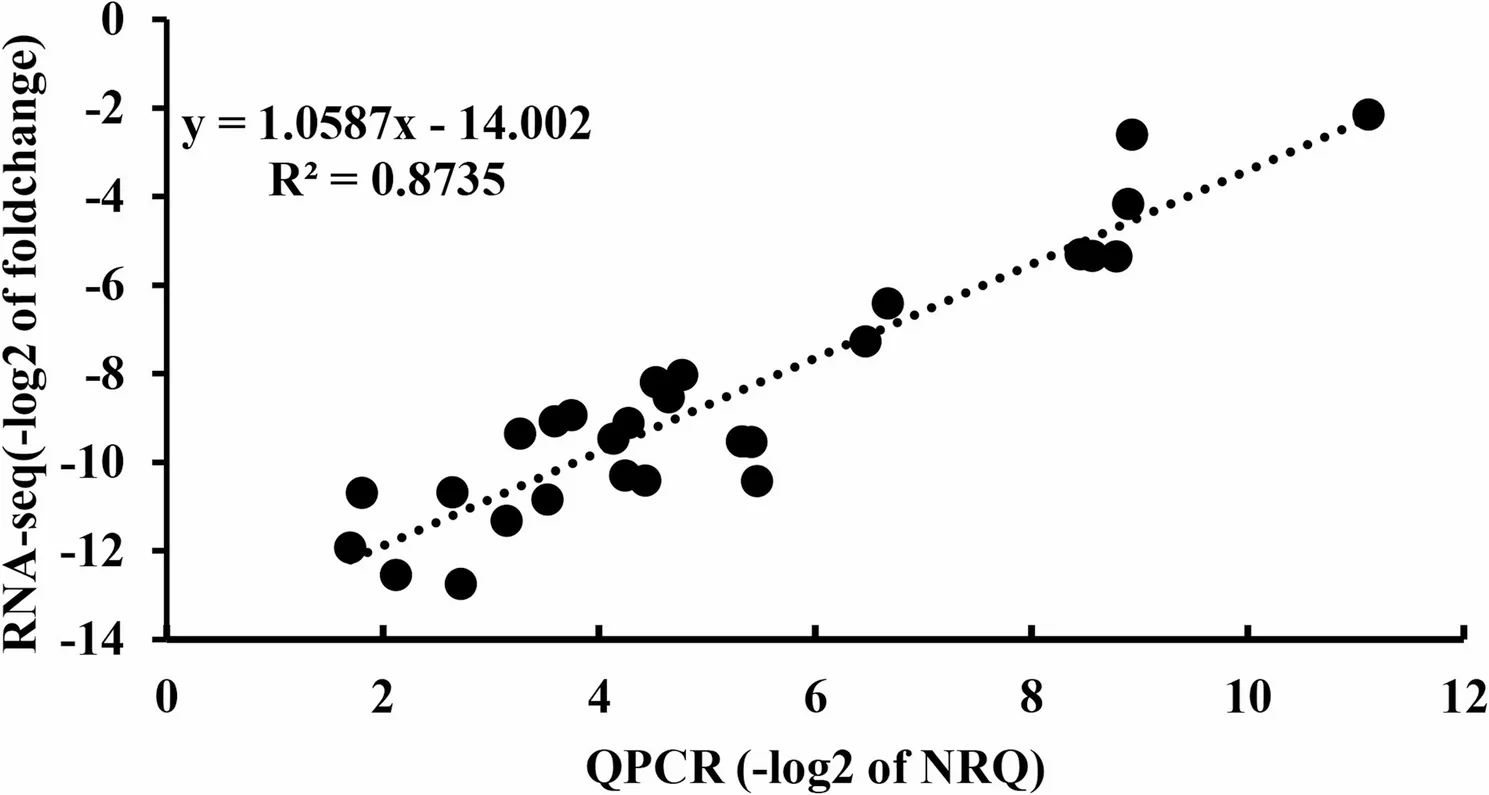
Validation of RNA-seq data by qPCR. Scatter plots represent the transcriptional changes revealed by qPCR analysis and RNA-seq. The Pearson correlation coefficient (R^2^) was 0.8735

Seven DEGs upregulated in hulled barley were selected for qRT-PCR analysis to verify the reliability of RNA-seq data (Figure S1). The qRT-PCR results were highly consistent with the RNA-Seq data (R^2^ = 0.8735), confirming the accuracy of the transcriptomic analysis (Fig. 7).

### Verification of the effects of metabolites

**Fig. 8:**
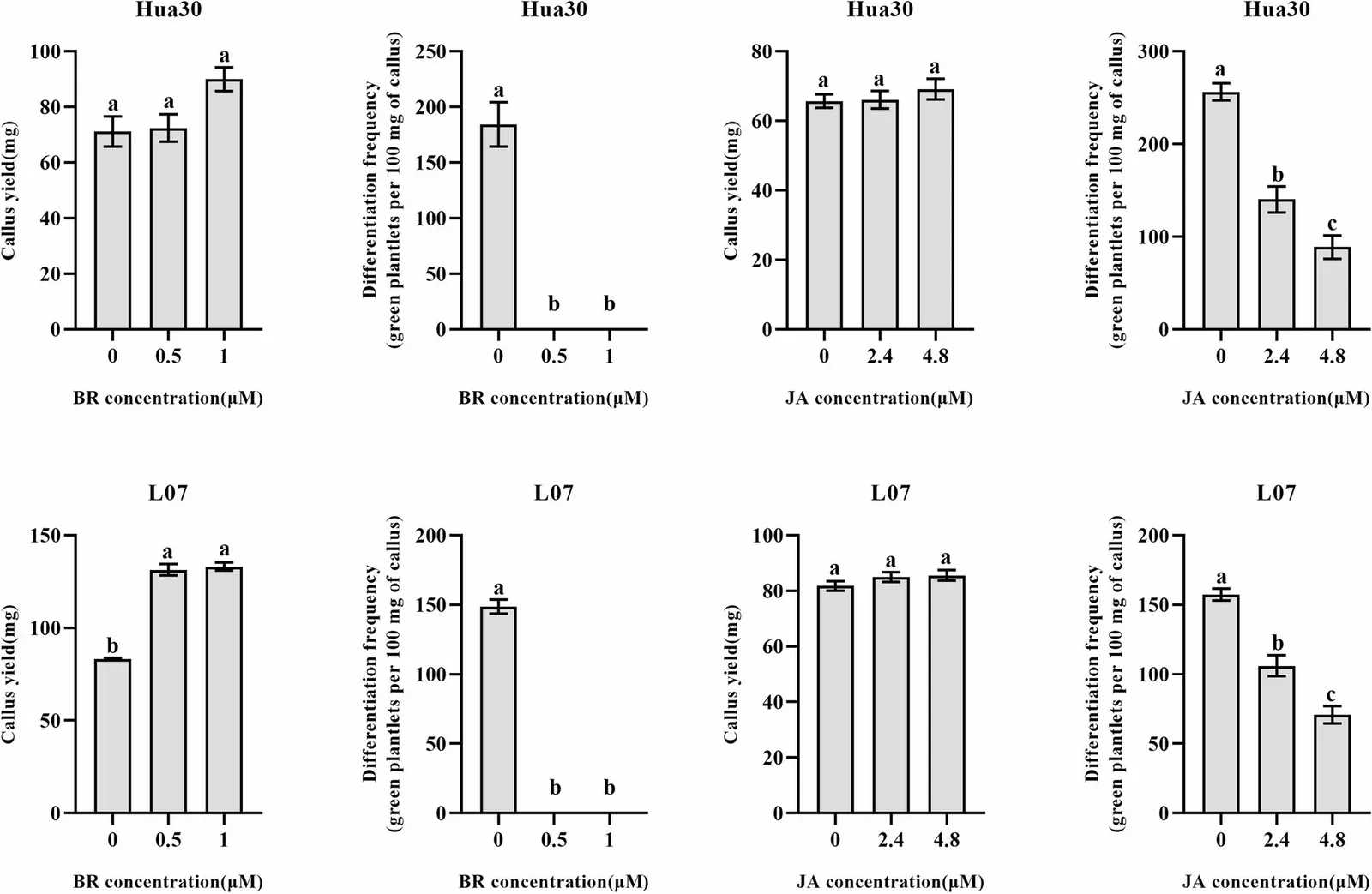
Effect of BR and JA addition in induction medium on callus yield and differentiation frequency of microspore culture in Hua30 and L07. The data were presented as means ± standard errors (*n* = 4). Different lowercase letters above the error bars indicate statistically significant differences based on one-way ANOVA by LSD test (*p* < 0.05)

Given the significant accumulation of JA and BR in the microspore-derived calli of hulless barley, these metabolites may influence the regeneration potential of the plant. Various concentrations of JA and BR were added to Hua30 and L07 during the callus induction stage, and the subsequent plant regenerative capacity was assessed (Fig. 8). The addition of BR to the induction medium increased the callus yield significantly, especially in L07; however, the resulting calli lacked regeneration capacity both in Hua30 and L07. Conversely, JA had a minimal effect on callus yield but caused a dose-dependent decrease in the differentiation frequency of green seedlings. These results indicate that high BR and JA concentrations in the callus severely impair the subsequent conversion to plantlets (Fig. 8).

## Discussion

IMC is a time-efficient and effective method for generating DH lines, making it a valuable tool in plant breeding programs [1]. Hulless barley, particularly highland barley, is an important cereal crop of the plateau regions, attracting attention for its superior nutritional and dietotherapeutic properties [18]. In previous studies, microspores from hulless barley formed calli, but these calli exhibited low regeneration capacity, whereas both callus formation and regeneration from hulled barley microspores were robust [17, 19]. This study further confirms these findings. Callus yields of hulless barley cultivars (XML22 and ZQ2000) were significantly higher than those of hulled barley cultivars (Hua30 and L07); however, almost no green plantlets were produced from hulless barley, whereas hulled barley displayed a differentiation frequency exceeding 200 green plantlets per 100 mg of callus. These differences in microspore-derived calli underlie the divergent plant regeneration capacities between hulled and hulless barley under comparable culture conditions. To investigate the molecular basis of this variation, integrated transcriptomic and metabolomic analyses were performed on microspore-derived calli. A total of 2,647 DEGs and 128 DAMs were identified in the calli of hulled barley. Integrated analyses revealed that plant hormone signal transduction was significantly enriched, demonstrating a key role for hormonal regulation in the enhanced regeneration potential of hulled barley microspore-derived calli.

The role of plant growth regulators (PGRs) is a central focus in the study of microspore embryogenesis [20]. However, most studies have concentrated on the effects of exogenous PGRs applied to culture media, with few addressing the influence of endogenous PGRs on microspore embryogenesis effectiveness [21]. Endogenous hormone levels and their balance are critical for microspore embryogenesis regulation [22]. Auxin is a key regulator of plant growth, development, and responses to environmental stress [23]. Auxin rapidly induces the expression of early auxin-responsive genes, including the AUX/IAA, GH3, and small auxin-up RNA (SAUR) families [24]. The transcriptional inhibitor Aux/IAA protein and the transcription factor ARF protein are central regulators of auxin signaling, whereas the GH3 gene modulates auxin homeostasis and mediates stress responses [25, 26]. Endogenous auxin participates in microspore reprogramming and in vitro embryo formation [20, 27]. In this study, three AUX/IAA genes, two ARF genes, and three GH3 genes were differentially expressed in microspore-derived calli of hulled barley, suggesting that hulled barley may possess a greater capacity to maintain auxin homeostasis during microspore embryogenesis.

CKs, together with auxin, are key regulatory factors in plant growth, development, and stress responses [28, 29]. Maintaining an appropriate auxin-to-cytokinin ratio is crucial for proper plant function [28]. Moreover, the endogenous hormonal balance between auxins and CKs has been shown to influence microspore embryogenesis, promoting androgenic structure formation and enhancing green plant regeneration capacity [21, 22, 30]. The ARR family of transcription factors primarily controls CK-mediated transcriptional responses, with B-ARRs acting as positive regulators and A-ARRs functioning as negative regulators. Distribution of ARR genes allows for fine-tuned regulation of growth, development, and stress responses via CKs [31]. In addition, B-ARRs can activate the transcription of WUSCHEL (WUS), which encodes key regulators of stem cell maintenance [32]. In this study, one B-ARR gene and six A-ARR genes were differentially expressed in microspore-derived callus of hulled barley, indicating their potential roles in the fine regulation of endogenous CKs during microspore embryogenesis.

ABA, a hormone involved in adaptive stress responses, plays a vital role in microspore embryogenesis [22, 33]. ABA enhances microspore embryogenesis and plant regeneration in *Brassica napus* L [33]. Springer et al. [22] reported that the absence of ABA-driven stress defense responses may contribute to the low efficiency of microspore embryogenesis induction in winter wheat microspore cultures. Although the initiation of microspore embryogenesis requires a certain ABA threshold level, accumulating evidence indicates that appropriate PGR homeostasis and crosstalk among auxins, cytokinins, and ABA are more important for effective microspore embryogenesis than the level of any individual PGR [30]. The role of ABA in microspore reprogramming remains complex. In addition to functioning as a stress-protective factor that enhances microspore viability during the induction of microspore embryogenesis, ABA-induced signaling cascades also regulate the activation of multiple genes, enzyme activities, redox states, and interactions with other PGRs [20, 34]. PYL receptors and PP2Cs constitute the core components of ABA signaling, and their regulation is critical for preventing excessive and harmful defense responses under abiotic stress conditions [35]. ABF is a key TF that regulates the expression of downstream ABA-responsive genes during ABA signal transduction [36]. Although ABA and abiotic stress signals suppress PP2C activity to activate stress responses, ABA and abiotic stress upregulate PP2C expression through ABF-mediated regulation, forming a feedback loop that maintains signaling homeostasis [35, 37]. In this study, one PYR/PYL gene, three PP2C genes, and one ABF gene were upregulated in hulled barley, indicating a coordinated reprogramming of the ABA signaling pathway that may help maintain hormonal homeostasis in hulled barley microspore-derived callus.

Ethylene is a gaseous phytohormone that regulates various developmental processes and responses to environmental stresses [38]. In the ethylene signaling pathway, one ETR gene, one EIN3 gene, and one ERF gene were upregulated in hulled barley. This pattern reveals that slight fluctuations in ethylene levels may trigger the signaling pathway during the specific developmental stage of microspore embryogenesis.

GAs are essential hormones that regulate nearly all aspects of plant development throughout the life cycle [39]. The addition of exogenous GA3 to the culture medium can enhance plant regeneration from microspore embryos [33, 40]. However, information regarding the roles of GAs in cell culture systems is limited [34]. In this study, one upregulated GID2 gene and one downregulated PIF gene were identified in microspore-derived calli when comparing hulled and hulless barley, indicating that endogenous GA signaling may participate in the regulation of microspore callus development.

SA is a key phytohormone involved in plant responses to both biotic and abiotic stresses and plays a crucial role in balancing plant growth and defense [41, 42]. NPR1, a SA receptor, regulates the transcription of SA-responsive genes, such as PR-1, through interactions with specific TFs of the TGA family, thereby activating immune responses [43]. In hulled barley, two downregulated genes (TGA and PR-1) were identified in the SA signaling pathway, indicating that endogenous SA signaling may be more closely associated with microspore callus development than with defense responses.

BRs are a type of plant steroid hormones that play essential roles in various processes of plant growth and development, including cell elongation and division, photomorphogenesis, xylem cell differentiation, and reproduction, as well as abiotic and biotic stress responses [44]. Brassinolide is the most biologically active form of BR [45]. JA is another versatile phytohormone that regulates stomatal function, leaf expansion, leaf senescence, and flower development [46]. Furthermore, JA is one of the most prominent hormones associated with plant resistance to both biotic and abiotic stresses [47]. BR and JA were significantly enriched in hulless barley. However, no DEGs were identified in the BR and JA signaling pathways in the microspore callus between hulled barley and hulless barley. One possible explanation is that transcriptional regulation of these signaling components occurred before the sampling time point examined in this study. Furthermore, several DEGs associated with JA and BR biosynthesis were identified in the alpha-linolenic acid metabolism and BR biosynthesis pathways. These DEGs may contribute to the reduced JA and BR levels in hulled barley.

JA can improve microspore embryogenesis and plant regeneration when applied at an appropriate concentration (1.0 mg·L^− 1^) and incubation duration (24 h) in *Brassica napus* L [48]. BR exerts a positive effect on microspore embryogenesis and plantlet regeneration in pakchoi, although the optimal brassinolide concentrations vary among different genotypes [49]. However, Ferrie et al. [50] demonstrated that BR increased microspore embryogenesis in *Brassica juncea*, but not in *B. carinata*, *B. nigra*, or *B. rapa*. Adding BRs to the induction medium did not affect plantlet regeneration. In this study, the JA and BR contents in the microspore-derived callus of hulled barley were lower than those in hulless barley. The roles of JA and BR in the induction and differentiation of microspore callus in hulled barley were further examined by applying different concentrations of these hormones during callus induction. Both JA and BR promoted the induction of microspore callus in hulled barley. However, no differentiation ability was observed in the microspore callus formed in the presence of BR, whereas the differentiation ability of callus induced with JA decreased as the JA concentration increased. One possible explanation is that the addition of exogenous BR and JA may accelerate the maturation of the microspore callus, thereby leading to the loss or reduction of its differentiation ability. Therefore, further investigation is warranted to determine whether the application of BR and/or JA inhibitors in microspore culture of hulless barley could enhance the regeneration ability of callus.

Microspore embryogenesis is an in vitro culture system in which haploid microspores are reprogrammed to follow the embryogenic developmental pathway in response to external stress [4]. TFs are pivotal regulators of gene expression and epigenetic reprogramming, enabling plants to adapt to diverse environmental and developmental signals [51]. Several key TF families, including WOX, BBM, and LBD, play essential roles in plant regeneration [52]. In this study, one WOX7 gene, one BBM2 gene, and two LBD genes were upregulated in the microspore-derived callus of hulled barley, demonstrating that their specific expression likely contributes to enhanced regeneration potential.

The *nud* gene encodes an ERF transcription factor homologous to the *Arabidopsis* WIN1/SHN1 gene and governs the hulled versus hulless caryopsis phenotype in barley [53]. The ERF transcription factor WIN1 was upregulated more than sevenfold in the microspore-derived callus of hulled barley compared with that in hulless barley. This finding indicates that the caryopsis phenotype may be closely associated to the regenerative capacity of the microspore-derived callus.

## Conclusions

Overall, this study presents a comprehensive multi-omics characterization of the molecular differences in microspore-derived callus between hulled and hulless barley. The analyses revealed that plant hormone signaling is a critical determinant of regeneration potential. Several TFs associated with plant regeneration exhibited differential expression patterns between hulled and hulless barley, highlighting their key roles in regulating regeneration capacity. These findings provide valuable insights into the molecular mechanisms underlying plant regeneration.

## Materials and methods

### Plant growth conditions

Hua 30 and L07 are two-rowed barley varieties. Hua30 is a released cultivar, whereas L07 is an elite breeding line. Both were provided by the Shanghai Academy of Agricultural Sciences (SAAS), Shanghai, China. Zangqing2000 (ZQ2000) and Ximala22 (XML22) are hulless barley cultivars supplied by the Tibet Academy of Agriculture and Animal Husbandry Sciences, Tibet, China. All cultivars were grown in experimental fields at SAAS under standard agronomic management practices. Irrigation and fertilization were applied according to routine field management protocols throughout the growth period.

### Microspore culture and plant regeneration

Microspores were isolated following the procedure described by Lu et al. [54]. Isolated microspores were resuspended in the induction medium and adjusted to a density of 1.0 × 10^5^ microspores/mL. One milliliter of the suspension was dispensed into 35 × 12 mm Petri dishes and cultured in the dark at 25℃ for 19 days until callus formation. A portion of the calli from each of the four cultivars was collected and immediately frozen in liquid nitrogen after 19 days of culture for subsequent transcriptomic and metabolomic analyses. The remaining calli were weighed and transferred to differentiation medium for regeneration of green plantlets. The total number of green plantlets was recorded using three biological replicates. The callus yield was defined as the total weight of callus produced per Petri dish after 19 days of culture. The differentiation frequency was calculated as the number of regenerated green plantlets per 100 mg of callus.

The induction medium was based on N6 basal medium supplemented with 90 g/L maltose, 0.5 mg/L Kinetin (KT), and 1.0 mg/L 2,4-dichlorophenoxyacetic acid (2,4-D), 0.4 g/L casein hydrolysate (CH), 1.6 g/L glutamine, and 0.976 g/L 2-(N-morpholino) ethanesulfonic acid (MES), adjusted to pH 5.8. The extraction buffer and differentiation medium were prepared according to Lu et al. [19]. The extraction buffer and induction medium were sterilized by filtration, whereas the differentiation medium was sterilized by autoclaving at 121℃ and 0.11 MPa for 18 min.

### Transcriptomic analysis

Total RNA extraction, cDNA library preparation, sequencing, and subsequent bioinformatic analyses were carried out by Novogene Co., Ltd. (Beijing, China). Three biological replicates were performed. Differentially expressed genes (DEGs) analysis was conducted by DESeq2 R package (v1.20.0) with an adjusted *p* value (padj) < 0.05 and absolute value of log2FoldChange ≥ 1. Gene Ontology (GO) and The Kyoto Encyclopedia of Genes and Genomes (KEGG) pathway enrichment analysis of DEGs were implemented by clusterProfiler R package.

### Metabolomic analysis

Metabolite extraction, Ultra-high-performance liquid chromatography–tandem mass spectrometry (UHPLC-MS/MS) analyses, data processing, and metabolite identification were performed at Novogene Co., Ltd. (Beijing, China). Six biological replicates were performed. Metabolites were annotated using the KEGG, Human metabolome database (HMDB), and LIPIDMaps databases. Differentially accumulated metabolites (DAMs) were identified in accordance with the following criteria: variable importance in projection (VIP) > 1 combined with *p* value < 0.05 and fold change ≥ 1.5 or ≤ 0.667. The KEGG database was utilized to conduct functional annotation and pathway enrichment analyses of these differential metabolites and metabolic pathways.

### Integrated metabolomic and transcriptomic analyses

Integrated analysis of metabolomic and transcriptomic datasets was conducted using the R software based on the identified DEGs and DAMs.

### Validation of Quantitative Real-Time Polymerase Chain Reaction (qRT-PCR)

Seven DEGs upregulated in Hua30 and L07 were randomly selected for qRT-PCR to validate transcriptome data. cDNA synthesis, qRT-PCR reactions, data analysis, and statistical evaluation were performed as described by Gao et al. [55]. The primers were designed using the Primer3 software. *HvActin* and *HvGAPDH* were employed as reference genes. The primer sequences for qRT-PCR were presented in Table S5.

### Verification of the effects of metabolites

Concentrations of JA and BR were applied during the microspore callus induction stage in Hua30 and L07. The calli were transferred to differentiation medium for plant regeneration after callus formation. The callus yield and differentiation frequency were recorded. Different concentrations of BR (0 µM, 0.5 µM, and 1 µM) and JA (0 µM, 2.4 µM, and 4.8 µM) were added into the induction medium respectively. The concentrations of BR and JA were set mainly based on Ferrie et al. [50] and Ahmadi et al. [48]. Each treatment was conducted in four biological replicates.

### Statistical analysis

All data were processed in Microsoft Excel 2013 and presented as the mean ± standard error. Significant differences (*p* < 0.05) were determined using one-way analysis of variance (ANOVA) combined with least significant difference (LSD) tests, performed in DPS software (version 7.05, China).

## Supplementary Information

Supplementary Material 1.

## Data Availability

The RNA-seq data have been deposited in the NCBI database under accession PRJNA1430978. The other datasets generated and analyzed during the current study are available from the corresponding author upon reasonable request.
